# Challenges and Opportunities for Recycled Polyethylene Fishing Nets: Towards a Circular Economy

**DOI:** 10.3390/polym13183155

**Published:** 2021-09-17

**Authors:** Rafael Juan, Carlos Domínguez, Nuria Robledo, Beatriz Paredes, Sara Galera, Rafael A. García-Muñoz

**Affiliations:** 1 LATEP, Polymer Technology Laboratory, Rey Juan Carlos University, Tulipán St., Móstoles, 28933 Madrid, Spain; rafael.juan@urjc.es (R.J.); nuria.robledo@urjc.es (N.R.); beatriz.paredes@urjc.es (B.P.); 2 GIQA, Group of Environmental and Chemical Engineering, ESCET, Rey Juan Carlos University, Tulipán St., Móstoles, 28933 Madrid, Spain; 3 REPSOL, Repsol Technology Lab, Agustin de Betancourt St., Móstoles, 28935 Madrid, Spain; sara.galera@repsol.com

**Keywords:** recycled fishing nets (rFN), recycled polyethylene, high-density polyethylene (HDPE), circular economy

## Abstract

Plastic waste generation has become an important problem that critically affects marine and oceans environments. Fishing nets gear usually have a relatively short lifespan, and are abandoned, discarded and lost, what makes them one of the largest generators of ocean plastic waste. Recycled polyolefin resins from fishing nets (rFN), especially from polyethylene (PE), have poor properties due to the presence of contaminants and/or excessive degradation after its lifetime. These reasons limit the use of these recycled resins. This work aims to study the incorporation of recycled fishing nets PE-made to different grades of virgin PE, in order to evaluate the potential use of these rFN in the development of new products. The recovered fishing nets have been fully characterized to evaluate its properties after the collection and recycling process. Then, different PE virgin resins have been mechanically blended with the recovered fishing nets at different recycling contents to study its feasibility for fishing nets or packaging applications. Critical mechanical properties for these applications, as the elongation at break, impact strength or environmental stress cracking resistance have been deeply evaluated. Results show important limitations for the manufacture of fibers from recycled PE fishing nets due to the presence of inorganic particles from the marine environment, which restricts the use of rFN for its original application. However, it is proved that a proper selection of PE raw resins, to be used in the blending process, allows other possible applications, such as non-food contact bottles, which open up new ways for using the fishing nets recyclates, in line with the objectives pursued by the Circular Economy of Plastics.

## 1. Introduction

Plastics have become ubiquitous materials in our lives with a wide range of applications thanks to their excellent properties and low cost of production. Recent reports estimate that around 50.7 million tonnes of plastics were demanded in Europe in 2019 [1]. These resins are used to manufacture a wide range of products, mainly in the packaging (39.6%) and construction (20.4%) sectors. However, with the exponential growth of plastic resins production, plastic pollution has become an important issue for countries and ecosystems around the globe, and especially for marine environments [2,3,4]. According to recent reports, is estimated that around 11 million metric tons of plastic debris ends into the oceans every year, and is expected that without additional measures or actions, this flow of plastics litter will nearly triple in 2040 (around 29 million metric tons per year) [5]. From this source of plastic waste, over 80% was originated from land, while the rest is related to marine activities [6]. Measures have been currently adopted by governments and industries to reduce plastic debris from land [7,8]. However, those related to marine activities are still a challenge due to the difficulty to collect this plastic waste and the mismanagement of discarded fishing gear.

In Europe, goods related to fishing activities (Abandoned, Lost or otherwise Discarded Fishing Gear—ALDFG) are listed among the 10 most common debris found on coasts and beaches (27% of total litter) [9]. Additionally, fishing goods have usually a short lifetime that together with their loss at oceans and seas produces that around 640,000 tonnes of fishing gear are discarded globally every year [10], although the underestimation about the real metrics of abandoned fishing gear could hinder the real impact of these source of contamination [11]. These debris lead not only to important effects on marine flora and fauna due to plastic consumption and “ghost fishing” [12,13], but also cause and striking economic and social harm [14]. To combat this concern and reduce the plastic pollution at marine environments, important resources are being employed to develop new ways to reduce, reuse and recycle these products [15], under the objective to achieve a Circular Economy and the guidelines adopted in this sense by the European Union Council [16].

Nevertheless, recycling of fishing goods is not an easy task, as they are made from different plastics, mostly from polyamide (PA), polyethylene (PE), and polypropylene (PP) [17]. In particular, high-quality polymers need to be used to manufacture these items owing to strict requirements of most fishing gear, specifically the fishing nets. Among these resins, recycling rates differs depending on their ease of recycling and availability. PA is extensively used, mainly for manufacturing of gill nets due to its toughness and elasticity. Discarded fishing nets from PA are currently both mechanical [18] and mainly chemically recycled via depolymerization [19]. Chemical recycling allows transforming nylon nets back into recycled materials, obtaining a yarn that could be used in the manufacture of clothing or in the development of several new products [20].

On the other hand, fishing nets from PE and PP polyolefins have a lower value, as both are usually used to produce trawl nets, which suffer from abrasive damage [21]. Another important concern that must considered by the recyclers is the habitual presence of organic matter [22], which requires an exhaustive cleaning step that increase the economic costs of the process. For this reason, recycled resins from these materials usually have a lower performance and makes them difficult to compete with their virgin counterpart [23]. Most research have focused on the use of these recyclates as reinforcements fibers for construction applications [24,25,26] since their use as a main material for the manufacture of new products with the same application has been previously rejected due to their poor mechanical performance [27].

Several companies are producing recycled PA, HDPE, and PP from fishing net materials, via mechanical recycling [28,29]. Even if a sorting and separation step is essential to improve the properties of the recycled resins obtained, mechanical recycling of fishing net could still be a viable way of using these recycled plastics in the industry. To improve their properties and extend their use as a recycled material, blending with raw resins at different percentages could guarantee the requirements for the desired applications. Blending of virgin and recycled materials was demonstrated to be a feasible strategy to reuse plastic resins and help the development of a Circular Economy of Plastic [30,31,32].

**Figure 1:**
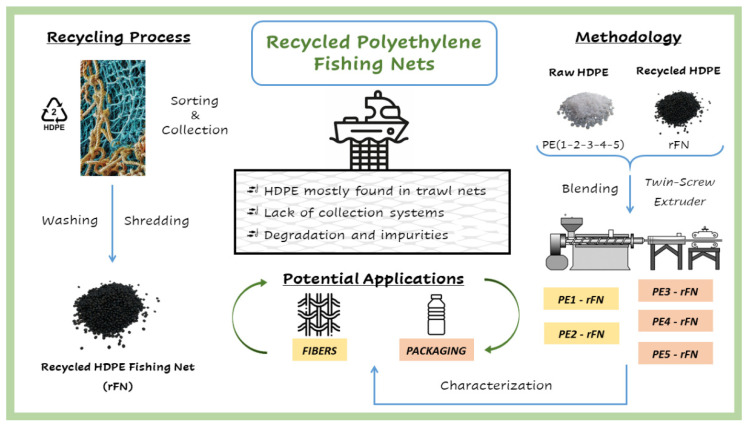
Flowchart of the experimental procedure.

The objective of this work is to study the incorporation of recycled polyethylene obtained from fishing nets to different grades of virgin PE (Figure 1). Although one of the aims is to obtain a blending material that can be used again in the manufacture of fishing nets, considering the properties of the recycled material, other potential applications related to bottle packaging were explored. The homogeneity and possible presence of contaminants in the recycled material were determined through Fourier Transform Infrared Spectroscopy (FTIR), Differential Scanning Calorimetry (DSC), Thermogravimetric Analysis (TGA) and Temperature Rising Elution Fractionation (TREF) measurements. These techniques, together with Gel Permeation Chromatography (GPC) and rheological measurements, were also used to study the chemical composition and homogeneity of all blends prepared. Mechanical properties were established by tensile, flexural and impact tests. The Environmental Stress Cracking Resistance (ESCR) of these blends was also evaluated to overall determine the long-term performance of these materials and their suitability for fishing nets and packaging applications.

## 2. Materials and Methods

### 2.1. Materials

Discarded HDPE fishing nets from Spanish harbors were sorted and separated from other polymers such as PA and PP to reduce cross contamination. Once collected, they were washed and cleaned, shredded, and extruded to obtain homogeneous pellets from these recycled HDPE fishing nets (rFN). This recyclate was blended with five raw resins used in different applications such as in fibers and packaging products. The blending process has been carried out in a Collin ZK 50 counter-rotating twin-screw extruder, containing up to 75 wt.% of rFN, and the corresponding raw resin for the remaining portion. The extrusion temperature goes from 185 to 240 °C with a screw speed of 60–80 rpm.

### 2.2. Molecular and Physical Characterization

To characterize the molecular structure of the resins, molecular weight (Mw), molecular weight distribution (MWD), and short chain branching (SCB) distribution were determined on a high temperature Gel Permeation Chromatography (GPC) GPC-IR6 (Polymer Char). The set of columns and infrared detector equipped on the GPC-IR6 allows distinguishing differences of 1 branch per 1000 atoms of carbon. Samples were dissolved in 1,2,4-trichlorobenzene (TCB) stabilized with BHT (2,6-di-tert-butyl-4-methylphenol) at a concentration of 0.75 mg/mL. The flow rate and temperature for the analysis was 1.0 mL/min and 160 °C, respectively.

TREF experiments were carried out on a commercial CRYSTAF-TREF instrument model 300 (Polymer Char). Samples of 80 mg were dissolved in TCB at 160 °C. After 60 min. of gentle stirring, the solution was injected into the column and held at 130 °C for 45 min. Then, the temperature decreased from 130 to 35 °C at a cooling rate of 0.5 °C/min to allow the polymer crystallization into the column. In the final step, the sample was eluted at a constant flow rate of 1 mL/min while rising the temperature from 35 to 140 °C. The polymer concentration was measured by an infrared detector and the crystallization curves were obtained from the first derivative.

Thermal measurements were developed at a heat rate of 10 °C/min with a DSC Mettler-Toledo 822e. Additionally, thermal stability of the recycled material was evaluated using a TGA/DSC 1 thermogravimetric analyzer (Mettler-Toledo), from 20 °C to 600 °C at a heating rate of 20 °C/min.

FTIR spectra were collected in attenuated total reflection (ATR) mode using a FT-IR Varian Excalibur 3100 spectrometer. The analysis was performed at room temperature and ambient humidity between 4000 and 400 cm^−1^, with 64 scans. The spectra were obtained in transmittance mode.

Rheological measurements were performed in a TA Instruments (model DHR-2) rheometer in the parallel plate mode. Sheets of around 2 mm of thickness have been previously compression molded and disks of suitable dimensions for rheological measurements have been obtained. The oscillatory viscoelastic determinations were carried out over the frequency range 0.01–100 rad·s^−1^. Deformation was set at around 1%, which corresponds to the linear viscoelastic region in all the pure polymers and blends, identified through previous amplitude sweeps. The measurements were performed at 190 °C.

Tensile and flexural tests were developed in a universal testing machine (MTS Alliance RT/5) at 23 °C and 50% relative humidity. Tensile tests were carried out using dumbbell shaped specimens 1BA at a crosshead speed of 50 mm/min, according to UNE-EN ISO 527-2:2012. Flexural and Charpy impact tests were performed on rectangular bars samples measuring 80 mm × 10 mm × 4 mm, extracted from a plate previously molded by compression. Flexural tests were carried out with a three-point bending geometry according to the ISO 178:2010 standard at a crosshead speed of 2 mm/min, while Charpy impact tests were done following the UNE-EN ISO 179-1:2010.

### 2.3. Environmental Stress Crack Resistance (ESCR)

Environmental stress cracking resistance was evaluated according to ASTM D 1693-15, under test condition B: Surface-active agent (Igepal CO-630) concentration of 10% volume in water at 50 °C. The tested materials have been previously obtained via compression molding.

## 3. Results and Discussion

The potential use of recycled HDPE fishing nets for different applications were evaluated through its blending with different raw resins. Table 1 summarizes the main physicochemical properties of the materials used in this work and the main application of the raw resins (fibers or packaging products).

Blends of virgin and recycled materials have been prepared using a counter-rotating twin-screw extruder to achieve a better homogenization and incorporating the recycled material at different rates (up to 75% of recycled resin). Each system prepared has been named according to the virgin resin and the recycled content (namely PEX-rFN-Y; where X corresponds to raw resin 1, 2, 3, 4 or 5; and Y corresponds to wt.% of recycled fishing net).

### 3.1. Blends for Fishing Nets Gear Application

The first objective of this study is to evaluate the potential use of these recyclates for the manufacture of fishing nets gear. Within this goal, two virgin resins have been chosen, named PE1 and PE2, respectively. These raw materials have been selected as they are typically used in the manufacture of fibers and nets, thus with similar properties to the recycled resin.

All blends were characterized by GPC and DSC to assess that blending process was successfully accomplished. Table 2 summarizes the results obtained for both systems (PE1—rFN and PE2—rFN). Both raw resins have a slightly higher Mw than rFN (Table 1). It is noteworthy that recycling process and degradation normally causes a drop in Mw for recycled materials [33]. Moreover, discarded fishing gear are greatly exposed to environmental conditions, being seriously affected by factors like ultraviolet light (UV) and temperature, which favors the degradation process [34]. The difference between the virgin PEs lies on their MWD and SCB distribution, being PE2 MWD slightly broader and with a higher SCB content than that of the PE1 resin. During the blending process, a progressive decrease in the Mw with the increasing of the rFN content is observed, for both systems. A broadening of the MWD is also observed, probably caused by the blending and extrusion process. Melting temperatures, for all blends prepared, were placed between the melting point of raw and rFN materials (140.0 and 139.5 °C for PE1 and PE2, and 138 °C for the recycled material). Moreover, the melting peak slightly shifted to lower temperatures with the increasing content of rFN. All these results indicate a good miscibility among the virgin and recycled materials, which confirms that the blending process was effective and a good compatibility among all polyethylene resins was achieved.

**Figure 2:**
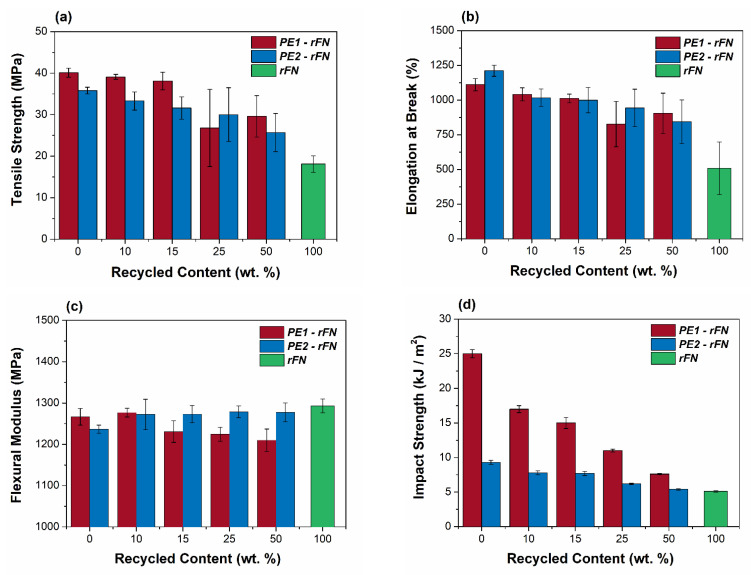
(**a**) Tensile strength, (**b**) Elongation at break, (**c**) Flexural modulus and (**d**) Impact strength resistance results for PE1—rFN and PE2—rFN systems.

After addressing the blend compatibility, the influence of the incorporation of rFN to virgin resins have been studied through the characterization of mechanical properties. Tensile stress-strain curves, flexural modulus and impact strength resistance were determined for both blend systems at room temperature (Figure 2a–d, respectively). At lower recycled contents (10 and 15 wt.%), tensile strength (Figure 2a) and elongation at break (Figure 2b) are little affected, with values close to those obtained for both raw resins. However, for higher recycled contents these properties are more affected compared to those of both virgin materials, with a greater dispersion of the measurements. rFN shows lower values and close to half of those observed for both raw resins. These facts could be explained due to the decrease in Mw with the increasing rFN content. Flexural modulus is less affected by the presence of the recycled net, with values between 1200 and 1300 MPa for all blends (Figure 2c). Finally, impact strength resistance is greatly affected by the incorporation of the recycled material, especially for the system “PE1—rFN”, which have a higher initial impact resistance than the system “PE2—rFN” that is closer to the rFN resin (Figure 2d).

**Figure 3:**
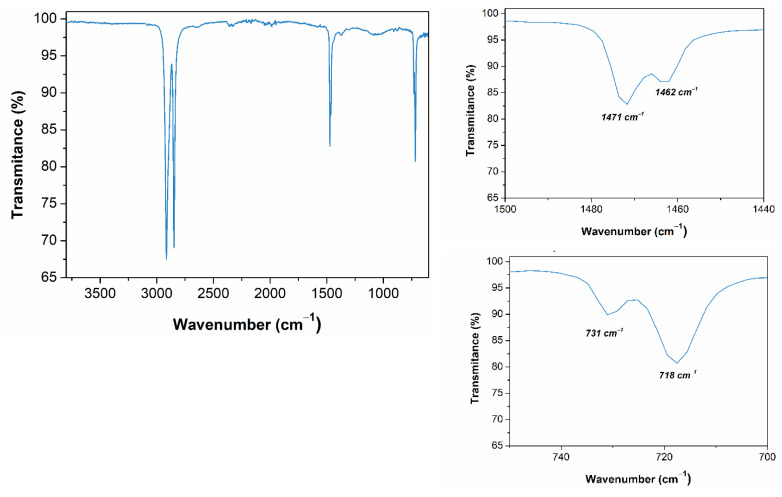
FTIR Spectrum of the recycled fishing net (rFN).

Moreover, cross contamination with other polymers or inorganic impurities could also be the cause of the premature rupture of the specimens during tensile tests since this is a common phenomenon for recycled polyolefins [35] that may also affect its long-term performance [36]. To confirm if the presence of unwanted substances could be the reason of the results obtained, further tests were performed for the rFN resin. Figure 3 shows the FTIR spectrum obtained for the rFN material. First, a doublet corresponding to asymmetric (2914 cm^−1^) and symmetric (2848 cm^−1^) stretching of CH_2_ are observed. Two more doublets are seen at 1470 and 725 cm^−1^, which correspond to bending and rocking deformations, respectively. All these peaks confirm the presence of PE [37]. No other signals are obtained in the spectrum, which could indicate that the separation from other fishing nets was effective.

**Figure 4:**
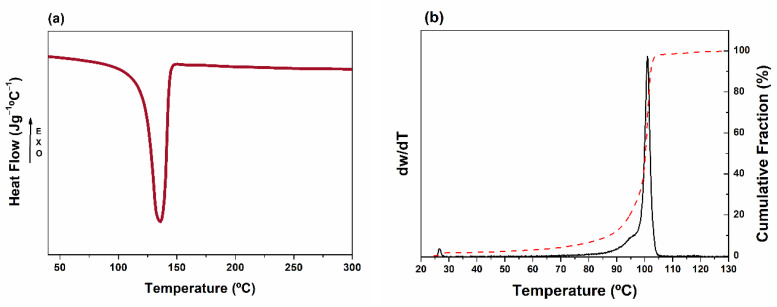
(**a**) DSC melting thermogram and (**b**) TREF curves for the recycled fishing net (rFN).

To further verify that cross contamination with other polymers did not take place during the recycling process, DSC and TREF analyses were performed (Figure 4). The DSC thermogram reveals a single melting peak at 138 °C, which corresponds to PE. No other melting peaks are observed, neither at 160 °C (PP) nor at 220 °C (PA-6) [38], which confirms the homogeneity of the recyclate and the successful previous separation process. However, DSC analysis is not really confident for the determination of PP contents under 2 wt.%, and TREF analyses were performed. TREF allows fractionating different polymer chains as functions of their crystallinity to understand the microstructure and the phase behavior of these materials. This technique, commonly used to determine the chemical composition distribution of different polymer blends, has recently proven to be more accurate for detection and quantification of PP impurities at low quantities than standard methodologies [39]. The thermograms reveals a main region between 90 and 105 °C composed by a very intense peak, which corresponds to the crystalline fraction of the HDPE chains (100 °C). The absence of other peaks, especially in the range of 120 °C, which corresponds to PP region, confirms that no or negligible cross contamination occurred during the recycling process [40].

**Figure 5:**
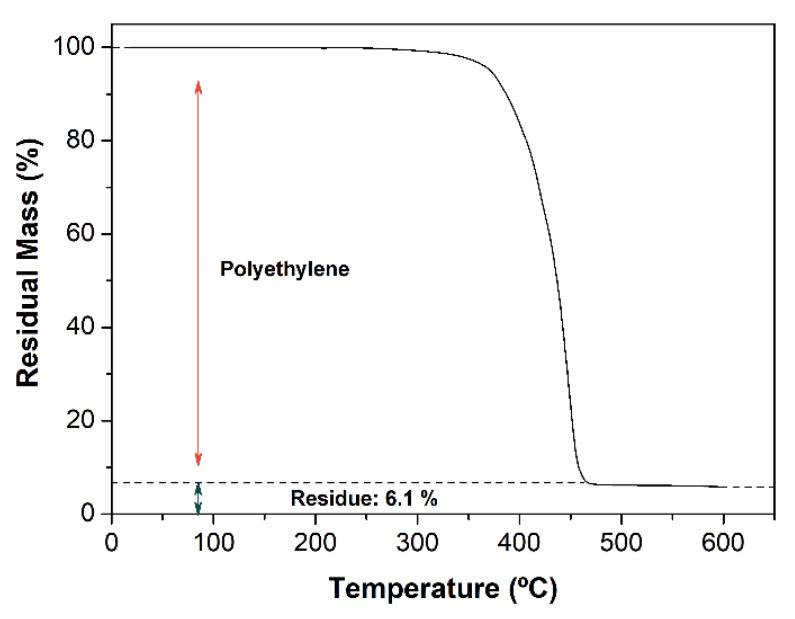
TGA curve for the recycled fishing net.

Finally, the thermal stability of rFN was evaluated through thermogravimetric analysis (Figure 5). The results show a residue around 6 wt.%, which could be assigned to the presence of CaCO_3_ (fillers). However, this residual high value in the sample also could indicate the presence of other inorganic contaminants (mainly silicates) present in marine environments. The presence of these impurities, even after the recycling process for the obtention of rFN, reveals the difficulty of washing and cleaning these kinds of recycled materials.

The presence of undesirable particles in the recycled samples may explain the results obtained during the mechanical characterization of both systems (PE1—rFN and PE2—rFN), as key properties such as elongation at break or impact strength resistance are greatly affected by the presence of contaminants. This fact clearly endangers its possible use in the manufacture of fishing nets gear since the presence of impurities could act as a stress riser and fracture initiator that may break the fibers during the manufacture and failing in the process of obtaining monofilaments and nets. As a result, rFNs do not seem suitable for the original application and strengthening us to search for other possible uses for this recycled material.

### 3.2. Blends for Packaging Application

The results attained for PE1—rFN and PE2—rFN ascertained its limited use in the manufacture of fishing nets, mainly due to the contamination present in the recycled resin. However, the overall properties obtained for the recycled materials could be useful for other applications. As previously mentioned, packaging industries are one of the biggest consumers of PE resins. The European regulations and objectives regarding Circular Economy of Plastics are expecting to incorporate recycled resins in packaging sector to reduce the raw plastic consumption in the next few years [41]. Within this aim, we have evaluated the recycled fishing nets as a possible recyclate source for the manufacture of non-food contact bottles, such as those used to contain cleaning or cosmetic products.

Three high-molecular weight blow molding raw HDPE resins have been chosen (named PE3, PE4 and PE5), in order to reinforce the expected loss of properties after blending with the recyclate. In this sense, the use of other additives or compatibilizers was avoided, as its use could increase the economic cost of the process hindering their recyclability [42]. Thermal and molecular characterization were carried out for all blending systems (PE3-4-5—rFN). Results are summarized in Table 3. All virgin resins have higher Mw and broader distributions than the rFN material. As the content of rFN increases the Mw of the blend decreases, and the distribution is becoming narrower. Both aspects suggest a good compatibility between both virgin and recycled resins, which is supported by the DSC curves that show a single melting peak for all systems (Table 3). Regarding the melting temperature, all blend systems appear to shift to higher temperatures with the increasing content of rFN, respecting the virgin PEs, which also implies good miscibility among the components.

**Figure 6:**
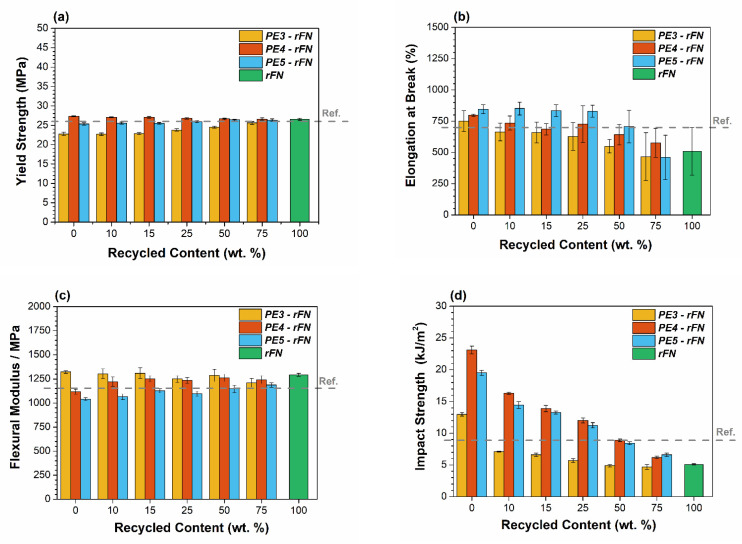
(**a**) Yield strength, (**b**) Elongation at break, (**c**) Flexural modulus and (**d**) Impact strength resistance results for PE3—PE4 and PE5—rFN blend systems.

For using in non-food contact bottles, the materials need to keep the stiffness and a high impact strength and environmental stress cracking resistances. For these reasons, tensile, flexural and impact test were performed for all blends. Additionally, the resistance to environmental stress cracking (ESCR) was measured to evaluate the influence of the recycled material into this critical parameter for packaging applications. The values obtained in all tests were compared with the standard values for common raw HDPEs used for packaging applications. Reference values indicated in Figure 6, as dot lines, were obtained as an average of different blow molding HDPE commercial bottles.

Figure 6a,b depicts a comparative of yield strength and elongation at break values obtained for all prepared systems. For yield strength and elongation at break, reference values are 26 MPa and 700%, respectively. Both properties are influenced by the incorporation of recycled resin into the blend, decreasing as the rFN content is higher, which results in the reduction of the Mw of the material. Nevertheless, for PE4 and PE5 systems contents up to 50% of recycled material makes the values remaining above or around the threshold. The higher Mw of both virgin resins allows incorporating more recycled material into the blend.

Regarding flexural modulus, the reference value must be closer to 1150 MPa to guarantee the minimum stiffness required for the bottles. As it can be seen in Figure 6c, all virgin and recycled resins have similar values. PE4 and PE5 have slightly lower rigidity, but with the increasing content of rFN the flexural modulus slightly increases. All blends with a 50 wt.% content of recycled material have values above the 1150 MPa, thus the stiffness is not a restriction for the use of this recyclate for packaging application.

Figure 6d also shows the impact resistance for the different blend systems analyzed. The impact strength is the most sensitive mechanical property to the recycled content and for the different blends is critically influenced by the low impact resistance of the rFN resin, around 5 kJ/m^2^. Minimum required impact resistance needs to be above 9 kJ/m^2^, value not reached for any blends of system PE3—rFN. The Mw of the raw resin critically mediates on the impact strength resistance of the systems, which rapidly decrease with the incorporation of rFN. However, results are improved when higher Mw resins such as PE4 and PE5, are used. Both systems allow reaching a recycled content of up to 50 wt.%, maintaining the minimum specifications. The higher Mw of PE4 and PE5 raw resins increases the overall impact resistance of the material, as a higher degree of entanglement and tie molecules density difficult the rupture of PE bonds and the fracture process, allowing the material to absorb more energy. The impact resistance falls dramatically with the increment of the recycled resin, as the rFN has almost half the Mw of the virgin resins. Additionally, degradation and inorganic impurities make the rFN more brittle, which lower the impact of the material after the blending process.

Finally, the environmental stress cracking resistance (ESCR) has been evaluated. This long-term mechanical property is crucial for the application considered, as in the presence of stress cracking agents, such as detergents or alcohols, PE suffers breakage after an interval of time [43,44]. Results for rFN show a low resistance to ESCR (~10 h). This recyclate has a low Mw, a property that greatly influences the environmental stress cracking resistance of PE materials. Moreover, the presence of inorganic impurities, previously detected, together with a certain level of degradation caused during the recycling process [45,46] also determines the lower ESCR performance of the rFN. On the other hand, raw resins PE3, PE4 and PE5 have overall higher ESCR values due to its high Mw, which helps to increase the tie molecular density [47,48,49].

**Figure 7:**
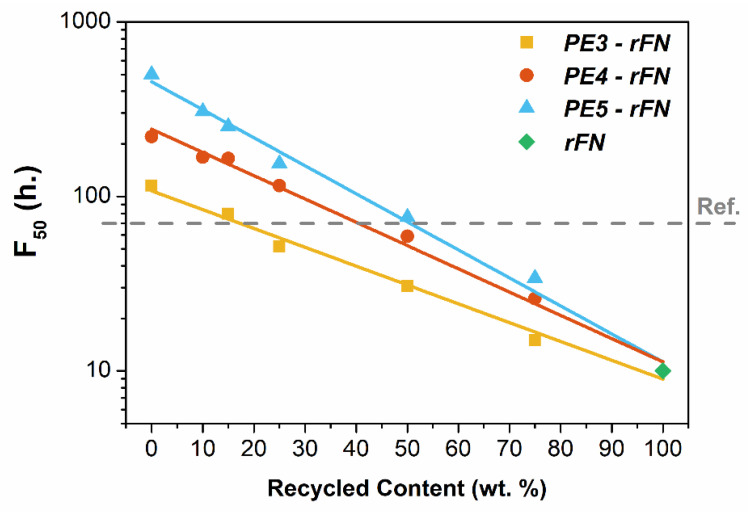
ESCR F_50_ values (h) vs. recycled content for all blends.

As shown in Figure 7, the same trend is observed for all blend systems. As the content of recycled resin increases the ESCR of each blend exponentially decreases, even at low contents of recyclate. When PE3 is used as a raw material, only 25 wt.% of rFN could be incorporated into the blend in order to fulfill the resistance for packaging applications, fixed at a threshold value of 70 h from commercial HDPEs used as reference. The loss of ESCR properties, promoted by the incorporation of rFN in the blends, can be compensated and commensurate with raw resins with higher Mw, such as PE4 or PE5 resins. Thus, higher ESCR values are achieved when PE5 is chosen as raw resin. This could be explained because of the higher SCB content of PE5 (Table 1), which in the case of the blends is mainly located in the high-molecular weight region, and therefore increasing the ESCR performance of the material [47]. In this way, it is possible to incorporate up to 50% of recycled resin in the PE5—rFN system, guaranteeing the standard value of the commercial reference materials (70 h).

Finally, for PE5—rFN system, which shows the best results in terms of mechanical and ESCR properties, a molecular model has been introduced to confirm the miscibility of the blend components from a morphological point of view. The double reptation model [50] for miscible blends, as expressed in Equation (1), is frequently applied to explain the viscoelastic behavior of polyolefin melts [51,52]. Indeed, rheology has become a potent tool for inferring morphological state.

(1) $$G\left(t\right)={\left[\underset{i}{\sum }\phi_{i\;}G_{i}^{1/c}\;\left(t\right)\right]}^{2}$$

**Figure 8:**
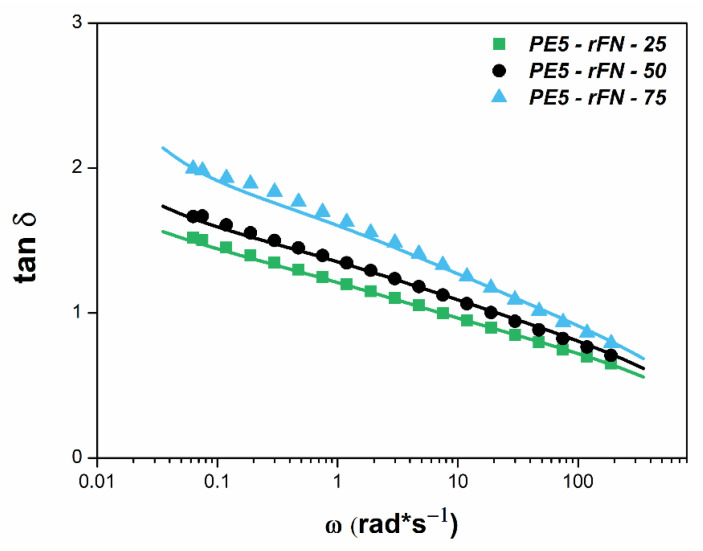
Tan δ versus the angular frequency for PE5—rFN system at 190 °C. Solid lines represent the results of the additive model for miscible blends.

Figure 8 represents the viscoelastic function tan δ, which is the ratio between loss and storage shear moduli (tan δ = *G*″/*G*′). As it can be observed, this additive model perfectly predicts the experimental response up to 50% of recycled content, which confirms the good miscibility among the PE5 and rFN, despite the differences in molecular weight of both resins. For PE5—rFN—75, a slight deviation of the model is observed, which can be attributable to the presence of impurities, which are more noticeable when the rFN is the main component in the blends.

Blends that incorporate virgin PEs with higher Mw enhance the mechanical performance of the recycled PE, even with the presence of slight contents of impurities or contaminants, which seems to be a good alternative for this kind of recycled HDPE from fishing nets. These results could be further improved with better handling and management of the discarded fishing nets, which would enhance the properties of the recycled material and helping to its better recyclability, even for different applications than its original use, and thus closing the loop on discarded PE fishing nets.

## 4. Conclusions

Plastic pollution is becoming an increasing problem in our world, with the marine environments especially affected by plastic debris. Discarded fishing nets, particularly those made of polyolefins, are subjected to harsh conditions that affect its properties, lowering its value as recycled materials. To evaluate possible uses for this recycling stream, rFN has been blended with different raw resins. First, blends with virgin HDPE were explored to be used again for the manufacture of fishing nets. This study produced poor results, despite the good compatibility exhibited between virgin and recycled resins. Mechanical properties showed a high scatter in the results, especially due to the inorganic contaminants detected in the rFN, which made more difficult the obtention of fibers with proper quality to satisfy the demanding requirements for fishing nets. This result dramatically decreases its use on the original application.

As other possible applications for the rFN, the use of this material in the manufacture of non-food contact bottles has been explored. To strength the properties of the recyclate, rFN was blended with three different high-molecular blow molding virgin resins, to ensure the minimum requirements for the packaging application. Results show that while some properties are less influenced by the recycled content, other key properties such as impact strength or environmental stress cracking resistances rapidly decrease. However, when using raw resins with remarkably higher Mw than rFN, as PE4 or PE5 resins, the incorporation of up to 50 wt.% of recycled material that meets the specifications was achieved. In this sense, it has been proven that is critical to know the properties of the recyclate to identify its possible uses. Additionally, a proper selection of virgin materials helps to compensate the loss of properties of the recycled materials and open up the way to new applications different from the original. The approach herein developed can help in the design of new products, as crucial step for the growth of a resilient and sustainable industry of plastics, in line with the objectives pursued by the Circular Economy.

## Figures and Tables

**Table 1:** Physicochemical properties of raw resins and recycled fishing net (rFN).

Material	Type	Mw (kg/mol)	Mw/Mn (−)	SCB/1000C	Tm (°C)
PE1	Fibers	149	6.94	1.3	140.0
PE2		146	8.42	2.1	139.5
PE3	Packaging	209	29.7	0.3	132.4
PE4		348	38.7	0.3	132.5
PE5		274	22.6	1.0	130.3
rFN	Recycled HDPE Fishing Net	121	8.40	2.5	138.0

**Table 2:** GPC and DSC results for PE1—rFN and PE2—rFN blend systems.

Blend System	% rFN	Mw (kg/mol)	Mw/Mn (−)	Tm (°C)
PE1—rFN	0	149	6.94	140.0
	10	147	7.17	140.2
	15	144	7.19	138.8
	25	139	7.56	137.9
	50	135	8.42	137.4
	100	121	8.40	138.0
PE2—rFN	0	146	8.42	139.5
	10	141	8.50	138.9
	15	141	8.93	138.5
	25	138	8.68	137.9
	50	132	9.00	138.1
	100	121	8.40	138.0

**Table 3:** GPC and DSC results for PE3—rFN, PE4—rFN and PE5—rFN blend systems.

Blend System	% rFN	Mw (kg/mol)	Mw/Mn (−)	Tm (°C)
PE3—rFN	0	205	22.1	132.4
	10	202	20.6	133.9
	15	201	20.1	133.5
	25	193	18.9	135.9
	50	175	15.1	136.7
	75	147	13.2	137.1
	100	121	8.40	138.0
PE4—rFN	0	348	38.7	132.5
	10	307	34.7	133.5
	15	307	33.2	134.1
	25	286	31.7	134.8
	50	255	27.1	135.7
	75	192	18.4	137.3
	100	121	8.40	138.0
PE5—rFN	0	277	21.7	130.3
	10	251	19.5	131.2
	15	250	19.3	132.3
	25	240	19.7	133.9
	50	205	15.2	135.1
	75	162	12.1	137.4
	100	121	8.40	138.0

## Data Availability

Not applicable.
